# Src- and Fyn-dependent apical membrane trafficking events control endothelial lumen formation during vascular tube morphogenesis

**DOI:** 10.1371/journal.pone.0184461

**Published:** 2017-09-14

**Authors:** Dae Joong Kim, Pieter R. Norden, Jocelynda Salvador, David M. Barry, Stephanie L. K. Bowers, Ondine Cleaver, George E. Davis

**Affiliations:** 1 Department of Medical Pharmacology and Physiology, University of Missouri School of Medicine, Dalton Cardiovascular Research Center, Columbia, MO, United States of America; 2 Department of Molecular Biology, UT Southwestern Medical Center, Dallas TX, United States of America; Johns Hopkins University, UNITED STATES

## Abstract

Here we examine the question of how endothelial cells (ECs) develop their apical membrane surface domain during lumen and tube formation. We demonstrate marked apical membrane targeting of activated Src kinases to this apical domain during early and late stages of this process. Immunostaining for phosphotyrosine or phospho-Src reveals apical membrane staining in intracellular vacuoles initially. This is then followed by vacuole to vacuole fusion events to generate an apical luminal membrane, which is similarly decorated with activated phospho-Src kinases. Functional blockade of Src kinases completely blocks EC lumen and tube formation, whether this occurs during vasculogenic tube assembly or angiogenic sprouting events. Multiple Src kinases participate in this apical membrane formation process and siRNA suppression of Src, Fyn and Yes, but not Lyn, blocks EC lumen formation. We also demonstrate strong apical targeting of Src-GFP and Fyn-GFP fusion proteins and increasing their expression enhances lumen formation. Finally, we show that Src- and Fyn-associated vacuoles track and fuse along a subapically polarized microtubule cytoskeleton, which is highly acetylated. These vacuoles generate the apical luminal membrane in a stereotypically polarized, perinuclear position. Overall, our study identifies a critical role for Src kinases in creating and decorating the EC apical membrane surface during early and late stages of lumen and tube formation, a central event in the molecular control of vascular morphogenesis.

## Introduction

Considerable advances have occurred over the past two decades in elucidating the molecular basis of vascular tube morphogenesis [1–7]. This resulted from joint studies *in vitro* and *in vivo* whereby key regulators have been identified and signal transduction cascades have been elucidated, which together control mechanisms underlying endothelial cell (EC) lumen formation [8–28] and downstream maturation events occurring following the recruitment of mural cells [15, 29–37]. To date, a key area that has been understudied is how ECs are apically and basally polarized during EC lumen and tube formation [11, 12, 20, 24, 38]. We have reported that small GTPases including Cdc42, Rac1, and k-Ras as well as the Ras and Rap effector, Rasip1, are recruited to subapical regions or apical membrane domains during lumen formation [11, 24, 38]. Furthermore, our previous work and that of others has revealed the involvement of the polarity regulators, Par6b, a Cdc42 effector, and Par3 in EC lumen formation [12, 28, 39]. In addition, we have also reported that cytoskeletal polarization occurs during lumen formation including marked subapical polarization of acetylated tubulin (which co-localizes with Cdc42 in subapical domains) and basal polarization of F-actin [21, 24, 38]. Post-translational modifications of tubulin including acetylation and detyrosination are strongly induced during EC lumen and tube formation and directly correlate with the ability of ECs to undergo tubulogenesis [21, 24]. Interestingly, previous studies indicate that vesicle transport mediated through kinesin motor proteins can be enhanced by tubulin acetylation [40]. Also, models of epithelial cell polarization and lumen formation have revealed the role of Rho GTPases (including Cdc42) and Rab GTPases such as Rab8a and Rab11a in the transport of vesicles that facilitate the development of apical domain [41–43]. Blockade of these molecules interferes with apical membrane polarization and the lumen formation process. Although there are distinctions between the polarization of ECs versus epithelial cells during lumen formation [5, 43], it will be important to approach such questions with regard to EC polarization/ lumen formation in a similar manner to that performed previously with epithelial cells.

In this report, we examine the role of Src kinases in apical membrane polarization during EC tubulogenesis in the context of vasculogenic or angiogenic tube assembly. We find the marked apical polarization of Src kinases, particularly Src and Fyn, during these processes. Both phosphotyrosine and phospho-Src are strongly enriched and polarized in apical membranes (including intracellular vacuole and apical luminal membranes) during EC lumen and tube formation *in vitro* and *in vivo*. Functional blockade of Src kinases strongly inhibits EC lumen formation during both vasculogenic tube assembly and angiogenic sprouting events. Activated Src kinases are localized in intracellular vacuole membranes that track along the microtubule cytoskeleton that is highly modified by acetylation. These fuse together in a polarized, perinuclear manner to create an apical membrane surface that carries and maintains activated Src kinases. We also demonstrated that expression of either Src-GFP or Fyn-GFP constructs in ECs target apically and stimulate EC lumen formation. Overall, this new work identifies a key role for Src kinases in controlling the development of the apical membrane domain as well as serving as a marker of this apical surface during EC lumen and tube formation.

## Materials and methods

### Reagents

Antibodies to acetylated tubulin and phospho-c-Raf-1 Y341 were from Sigma (St. Louis, MO). Antibodies directed to phospho-Erk1/2, Src, Fyn, Yes, Lyn, phospho-Src Y416, phospho-Pak2 Ser141 and phosphotyrosine were from Cell Signaling Technology (Danvers, MA). Antibodies to CXCR4 and ESM-1 were from R&D Systems (Minneapolis, MN). Alexa fluor® 633 phalloidin was from Molecular Probes (Eugene, OR). PP2 and PP3 were from Calbiochem (La Jolla, CA). Rat tail collagen type I was prepared as described previously [8].

### Cell culture

Human umbilical vein endothelial cells (HUVEC) were purchased from Lonza and were cultured and used from passages 2 to 6 as described [44]. For adenoviral gene expression experiments, ECs were infected for 4–5 h at 37°C (5% CO_2_) and after that time, endothelial growth medium was added. In many experiments, ECs were placed in collagen matrices as described and were incubated for 24 hr [44]. In some experiments, we utilized a defined growth factor system as described [33] to investigate lumen formation during vasculogenic tube assembly or angiogenic sprouting as well as apical membrane targeting in EC only or EC-pericyte co-cultures. Cultures were fixed with 3% glutaraldehyde and stained with 0.1% toluidine blue in 30% methanol prior to photography, visualization, and quantitation of tube areas by Metamorph software (Molecular Devices, Sunnyvale, CA).

### Transfection of ECs with siRNA

*Si*-RNAs were obtained from Invitrogen (Carlsbad, CA) or Ambion (Foster City, CA). *Si*-RNAs were transfected into cultured endothelial cells as previously described [44]. The *si-RNA* sequences are as follows: *Src si*-*RNA*, 5’-AAGUCAGAC ACUGAGAGGCAGUAGG-3’ and 5’-CCUACUGCCUCUC AGUGUCUGACUU-3; *Fyn si*-*RNA*, 5’-AUAGAAAGUGAAUAGGCACCUUUGG-3’ and 5’-CCAAAGGUGCCUAUUC ACUUUCUAU-3; *Yes si-RNA*, 5’-GAGAAUCUUUGCGAC UAGAGGUUAA-3’; *Lyn si-RNA*, 5’-CAGAAGAUUGGAGAAGGCUUGUAUU-3’ and 5’-AAUACAAGCCUUCUCCAA UCUUCUG-3’; *Control si-RNA*, (AM4637) Silencer Select Negative Control #2.

### Immunostaining and immunoblot analysis

For 2D staining, ECs were infected Src-GFP or Fyn-GFP virus and suspended in collagen matrices. After 6 h, ECs undergoing lumen formation were harvested by digesting the matrix with pre-warmed 1 mg/ml of high-purity collagenase (Sigma-Aldrich Corp., St. Louis, MO) in 37°C water bath. And then ECs were mixed with media and rapidly plated onto glass coverslips which were precoated with collagen. ECs were allowed to attach for 15 or 20 min at 37°C. ECs were fixed with 2% paraformaldehyde, permeabilized with 1% of Triton X-100, and stained for the indicated antibodies and stained cells mounted in slow anti-fade medium (Molecular Probes). For 3D staining, ECs or adenovirus expressing GFP infected ECs were suspended in collagen matrices and incubated at 37°C. Collagen gels were fixed with 2% paraformaldehyde for 1 h and treated with 1% Triton X-100. After blocking, primary antibodies were added to cultures overnight at 4°C. Cultures were repeatedly washed and then incubated with secondary antibodies. Final washes were performed over several hours. The stained cultures were imaged by confocal microscopy. For western blot analysis, 3D collagen gels were lysed using sodium dodecyl sulfate sample buffer, and immunoblot analyses were performed.

For the *in vivo* staining experiments, fixed mouse tissues were washed with phosphate-buffered saline (PBS), cryoprotected in 30% sucrose overnight, embedded in Tissue-Tek O.C.T. and sectioned. Sections were washed with PBS and blocked (1h RT 5% serum). Primary antibody incubations were performed at 4°C overnight (1:100 pSrc: Millipore, Billerica, MA); 05–677 L GFP: Aves (Tigard, OR); pTyrosine: Cell Signaling Technology; 9411S). Slides were washed with PBS, incubated in secondary antibody (4 h, RT). Slides were washed with PBS and mounted using Prolong Gold Mounting Medium with DAPI. Images were obtained using a LSM510 or LSM710 Meta Zeiss confocal.

### Generation of adenovirus

GFP fused Src family clones were amplified from human cDNA clones (Addgene) and subcloned to pShuttle vector using the primers: Src-GFP sense (5_- AGGTCGACGCC ACCATGGGTAGCAACAAGAGCAAG-_3), Src-GFP antisense (5_-AGTCTAGATCACT TGTACAGCTCATCCATGCCGTG-_3), Fyn-GFP sense (5_- AGGTCGACGCCACCAT GGGCTGTGTGCAATGTAAG-_3), Fyn-GFP antisense (5_-AGTCTAGATCACTTGTAC AGCTCATCCATGCCGTG-_3). Adenovirus for GFP-Rac1 and GFP-Rac1V12 were previously described [11]. A mCherry-α-tubulin clone was purchased from Addgene and adenoviruses were generated. Details of our adenoviral production protocol were carried out as previously described [11].

### Microscopy and data analysis

Image acquisition of EC lumen and tube-formation assays were done using an inverted microscope (Eclipse TE2000-E; Nikon CKX41) with dry objective 15x (NA 1.2). Image analysis was done using MetaMorph software (Molecular Devices, Sunnyvale, CA) by tracing the EC-lumen and tube area of individual high-powered fields of culture assays. We performed data analysis by using Microsoft Excel and Prism (GraphPad, San Diego, CA) to perform statistical analysis. Statistical analysis was completed using Microsoft Excel. Statistical significance was set at minimum with p< 0.01. Student t-tests were used when analyzing two groups within individual experiments (with a minimum n = 10). For confocal microscopy, ECs in collagen I matrices were imaged on a laser-scanning confocal microscope (Leica TC5 SP5) connected to a multiphoton system (Leica, Buffalo Grove, IL) using excitation wavelengths of 488 nm and 633 nm. The resolution images were captured using the 633 water immersion objective 63x (NA 1.2).

### Ethics statement

All animal studies were performed in accordance with UT Southwestern Medical Center Institutional Animal Care and Use Committee (IACUC) approved protocol APN 2008–0310, approval date September 25, 2014. Mice are housed in a modern air-conditioned facility and are maintained according to NIH guidelines. In addition, the facilities are supervised by full time veterinarians and technical staff and are fully accredited by the American Association for Accreditation of Laboratory Animal Care. Mice were rapidly euthanized by CO_2_ gas and asphyxiation which minimizes pain and discomfort and this method is consistent with the recommendations of the AVMA Panel of Euthanasia.

## Results

### Phosphotyrosine-containing proteins are enriched in the developing apical membrane surface of endothelial cells during tube morphogenic events

**Fig 1:**
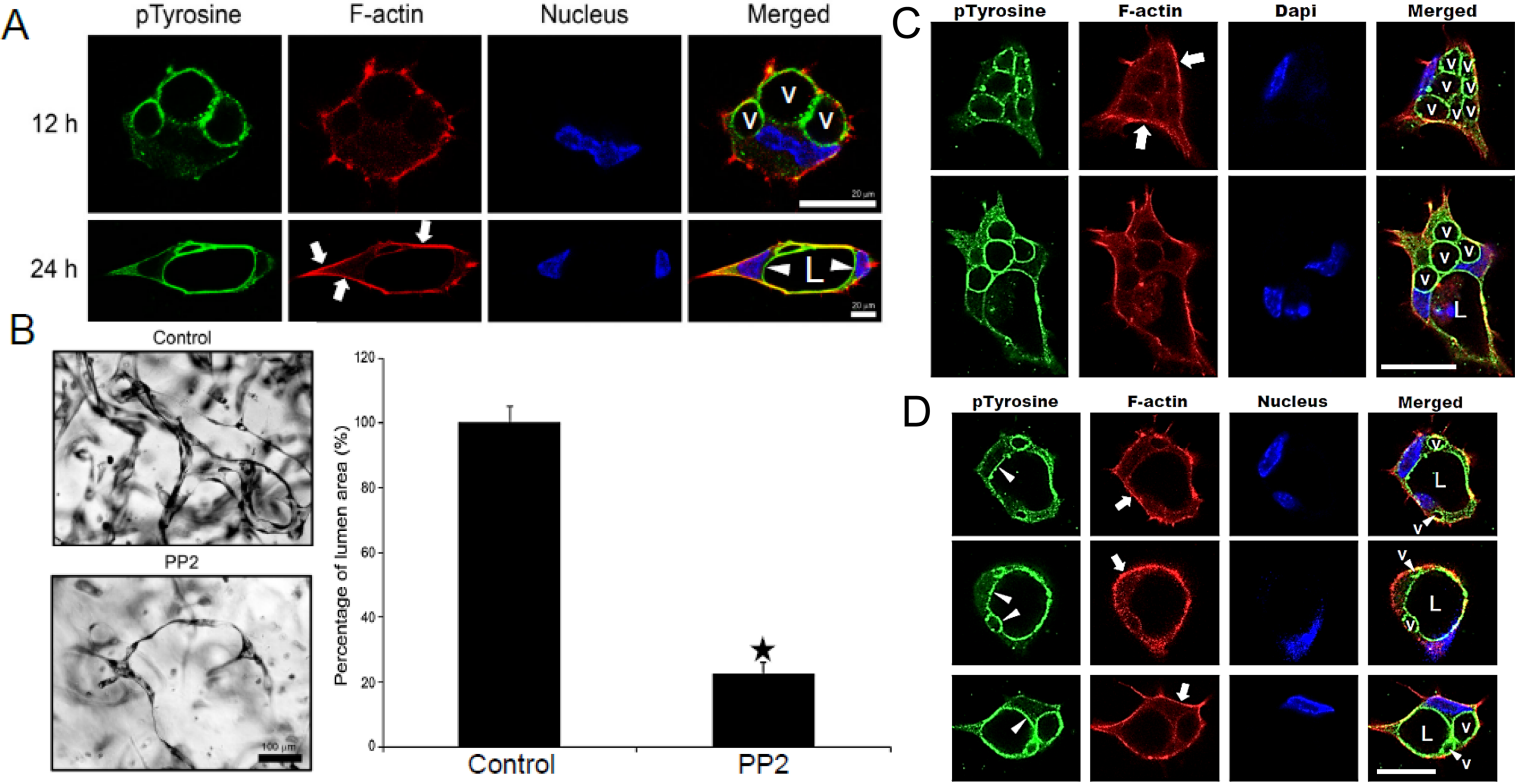
Marked accumulation of phosphotyrosine-containing proteins in the apical membrane domain of endothelial cells forming lumens and tubes. (A) ECs were suspended in 3D collagen matrices and after the indicated times were fixed and immunostained with anti-phosphotyrosine antibodies. Cells were also stained for F-actin using AlexaFluor-conjugated phalloidin, Hoescht dye to label nuclei and were imaged using confocal microscopy. Bar equals 20 μm. (B) ECs were seeded in 3D collagen matrices in the presence or absence of the Src inhibitor, PP2 at 10 μM. After 24 hrs, cultures were fixed, stained with toluidine blue and quantitated for lumen formation. Data are reported as the mean total vessel area per high-power field (HPF) ± standard deviation (SD) (*n* = 15, p < 0.01). Asterisks indicate significance compared to control cultures. Bar equals 100 μm. EC cultures were established and were fixed at 12 hr (C) or 16 hr (D) and were stained in the same manner as described in (A). Arrowheads indicate apical membrane staining; arrows indicate the basal distribution of F-actin staining. L indicates lumen, while v indicates intracellular vacuoles. Bar equals 25 μm.

**Fig 2:**
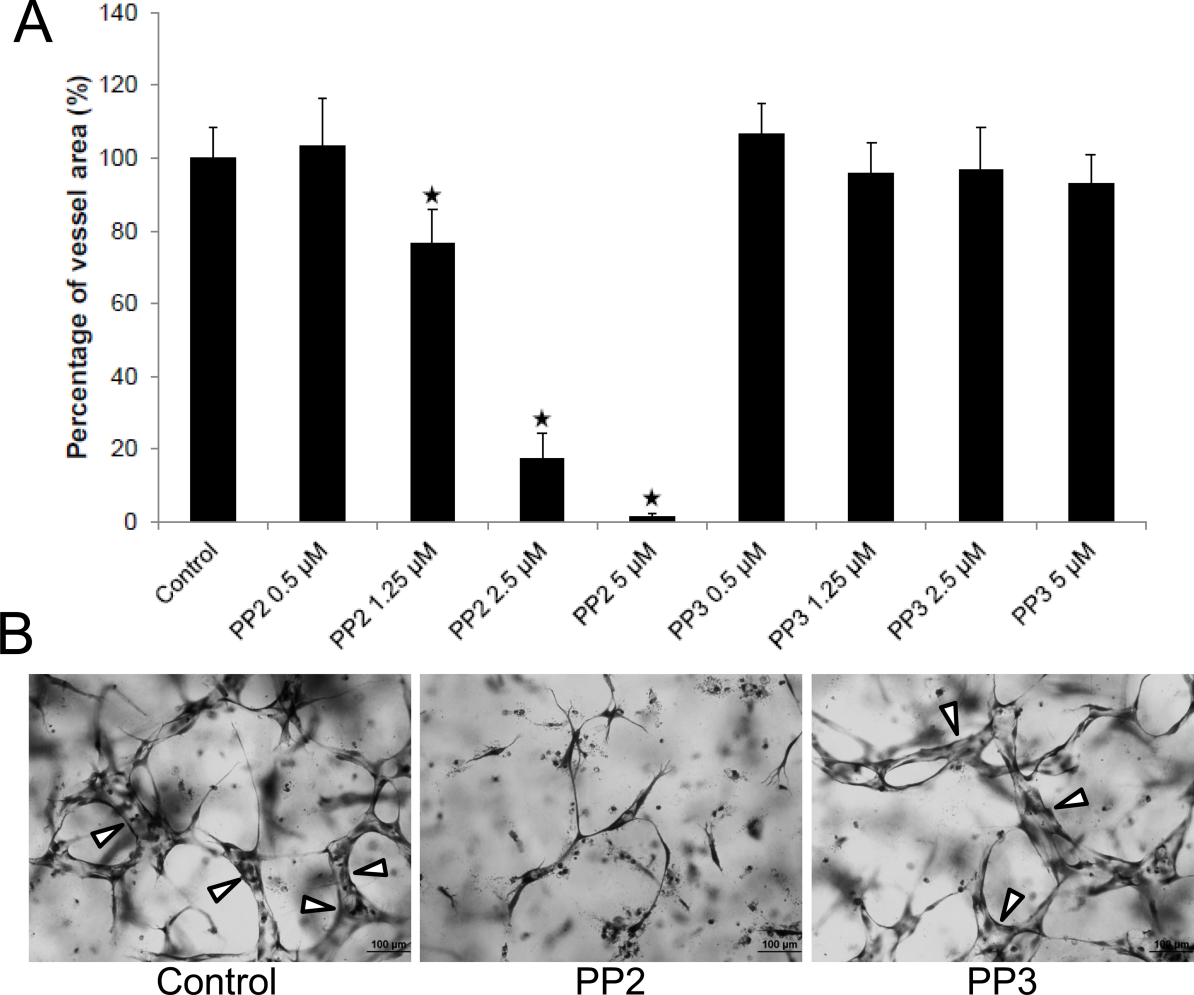
Src kinase activity is required for EC lumen and tube formation during vasculogenic tube assembly. EC cultures were established in the presence of varying doses of the Src inhibitor, PP2, versus an inactive PP3 control. After 72 hr, cultures were fixed, stained with toluidine blue, photographed (B) and were quantitated for tube area (A). White arrows indicate EC tubes. Data are reported as the mean total vessel area per high-power field (HPF) ± standard deviation (SD) (*n* = 15, p < 0.01). Asterisks indicate significance compared to control cultures. Bar equals 100 μm.

Previous work from our laboratory has revealed a role for Src family kinases (SFKs) in EC tubulogenesis and lumen formation [13], although the mechanisms that underlie their role remain undefined. Here we examine if Src kinase activities regulate EC apical-basal membrane polarity during EC lumen formation. EC cultures undergoing lumen formation were immunostained with anti-phosphotyrosine antibodies to look at downstream targets of SFKs at different times of the process. One of the intermediate steps in EC lumen formation is the formation of intracellular vacuoles which fuse together in a perinuclear, polarized manner to facilitate the rapid lumen formation process [2]. We demonstrate that strong localization of phosphotyrosine is observed in intracellular vacuole membranes and developing apical membrane surface, while F-actin is seen in a polarized, basal distribution (Fig 1A and 1C). Over time, the vacuoles fuse together to form the apical membrane, which is strongly labeled with phosphotyrosine antibodies (Fig 1A and 1D). To test for a functional role for Src during these processes, we added the Src inhibitor, PP2, and found that it completely blocks EC lumen formation (Fig 1B). PP2 inhibits EC lumen and tube formation in a dose-dependent manner, while its control, PP3, has no effect on this process (Fig 2).

**Fig 3:**
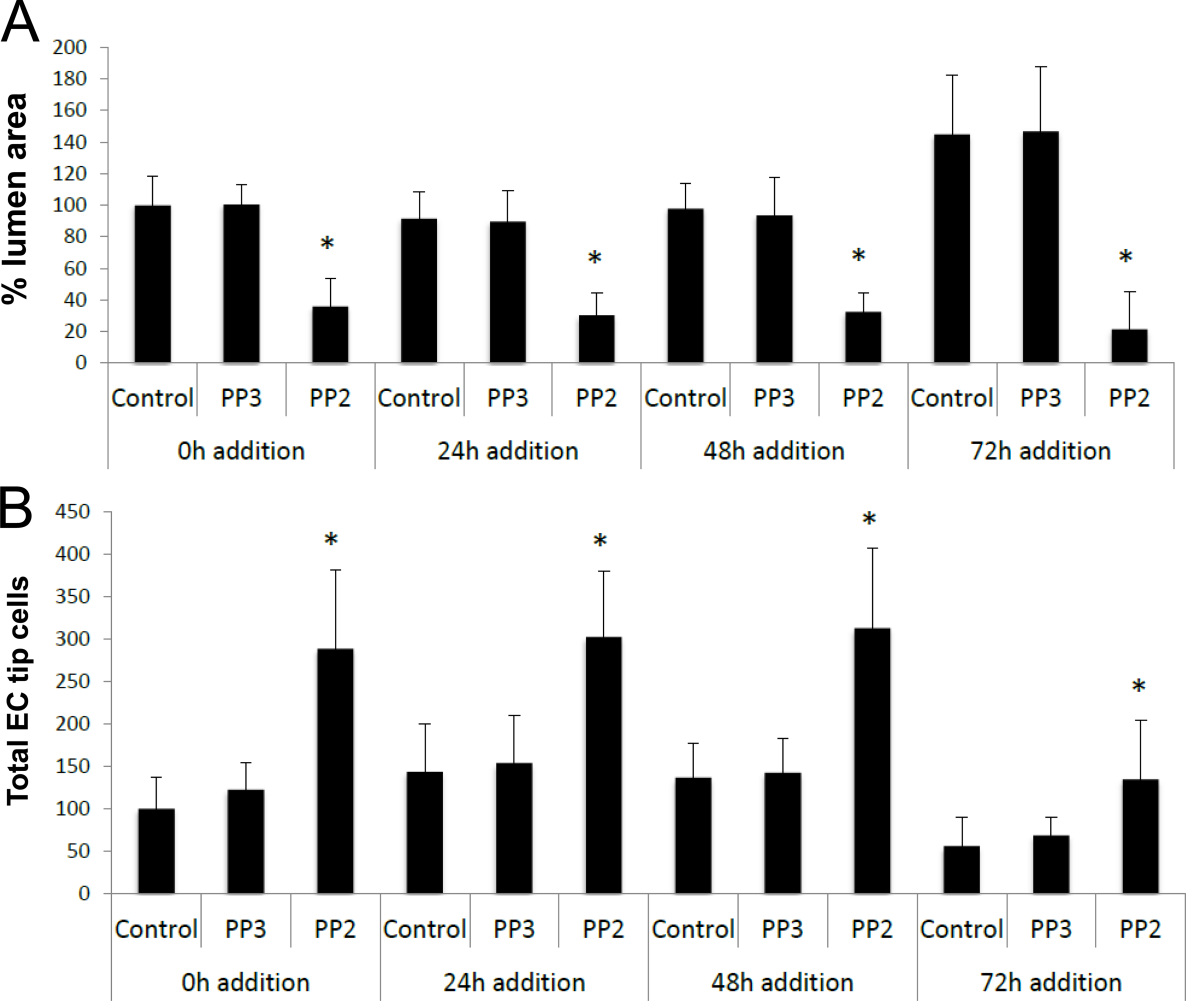
Src kinase activity is necessary for EC lumen formation during invasion and sprouting. (A,B) ECs were induced to invade and sprout and at the indicated times, PP2 or PP3 were added at 5 μM versus control. Cultures were fixed at 72 hr (for the 0, 24 and 48 hr addition points) or 120 hr (only for the 72 hr addition time point), stained with toluidine blue and quantitated for tube area (A) or EC tip cell number (B). Data are reported as the mean total vessel area per high-power field (HPF) ± standard deviation (SD) (*n* = 15, p < 0.01). Asterisks indicate significance compared to control cultures.

**Fig 4:**
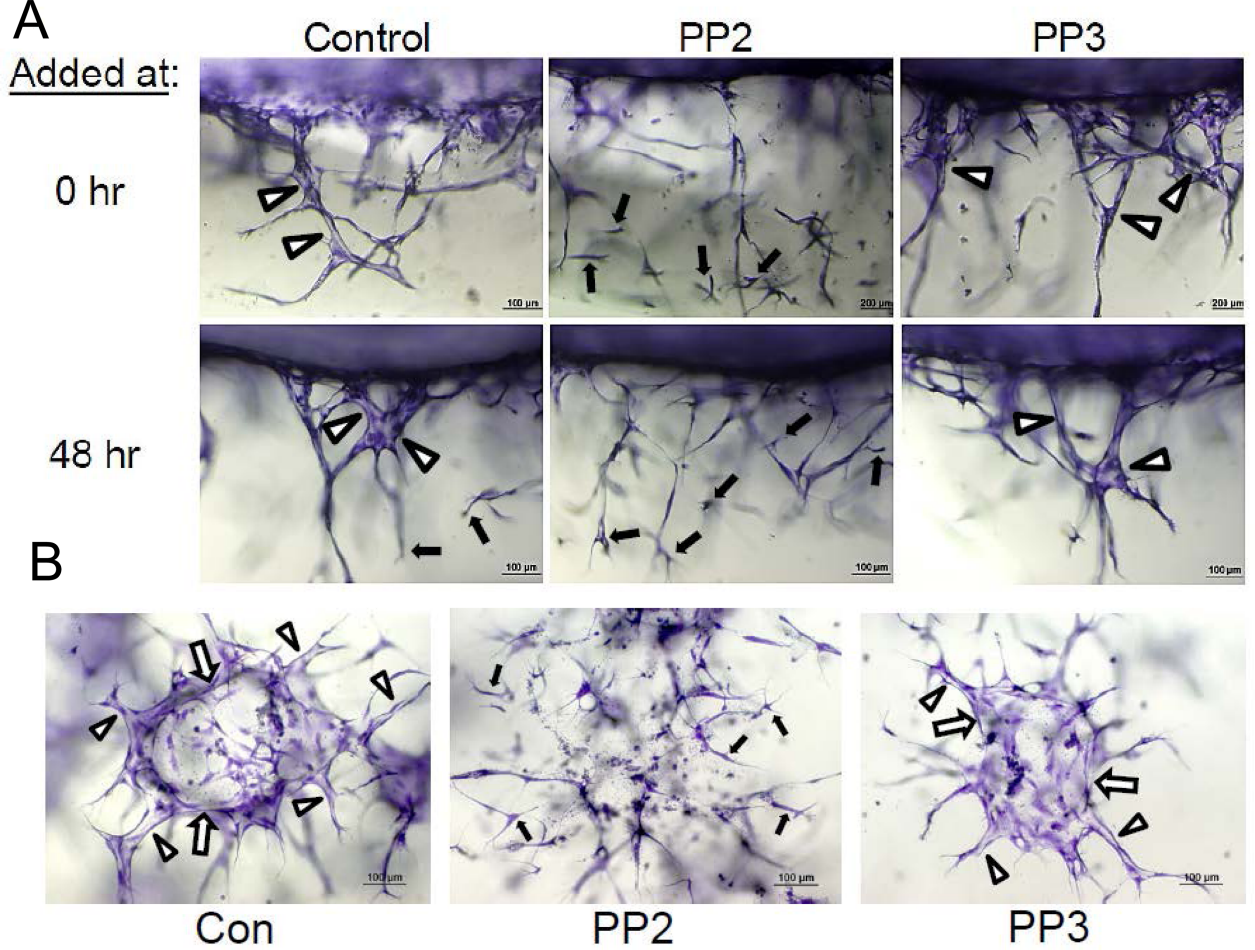
Src kinase activity is necessary for EC lumen and tube formation during sprouting from EC monolayer surfaces or EC-EC aggregates. (A) Photographs of cross-sections of EC sprouting cultures from a monolayer surface are shown at 72 hr following addition of PP2 or PP3 at 0 or 48 hr of culture. White arrowheads indicate EC lumens; black arrows indicate EC tip cells. Bar equals 100 or 200 μm. (B) EC aggregates with 15–25 ECs per aggregate were seeded in collagen matrices in the presence of PP2 or PP3 versus control. White arrows indicate the border of multicellular EC lumens; white arrowheads indicate EC lumens and tubes that have formed from EC sprouts; and black arrows indicate EC tip cells that are markedly increased in PP2-treated cultures secondary to inhibition of lumen and tube formation. Bar equals 100 μm.

There are two major ways that ECs form tubes during development which are through vasculogenesis and angiogenesis. The assays illustrated in Figs 1 and 2 demonstrate lumen and tube formation from ECs seeded as individual cells that assemble into tubes together, mimicking the process of vasculogenesis. We addressed whether blockade of Src activity would interfere with lumen formation using assays that mimic angiogenic sprouting (Figs 3 and 4). We show that PP2, but not PP3, blocks EC lumen formation when added at different time points during the sprouting process (Fig 3). Interestingly, ECs invade collagen matrices under these conditions quite readily even if lumen formation is blocked with PP2 (Fig 3). The cells that invade resemble EC tip cells, and in fact, blockade of lumen formation with PP2 leads to marked increases in tip cells compared to control conditions (Fig 4A and 4B). These EC tip cells can be immunostained with known markers of tip cells including CXCR4 and ESM1 (S1 Fig) [45, 46]. To demonstrate this phenomenon using a different assay model, we have seeded EC-EC aggregates and allowed sprouting and lumen formation to occur over 24 hr in the presence of PP2 vs. PP3 control. The initial aggregate forms a lumen structure (the borders indicated by white arrows) and sprouts with lumens can be observed in the control and PP3 treated cultures (Fig 4B). In contrast, PP2-treated aggregates demonstrated disassembly of the lumen with marked sprouting of EC tip cells, but without apparent lumens or tube structures (Fig 4B). This result is completely consistent with what we observed in the invasion assay system from a monolayer surface (Fig 4A). Another point is that PP2 addition (after 48 or 72 hr) to cultures that have lumens and tube structures (Fig 4A), leads to collapse of these lumens and tubes (i.e. tube disassembly) (Fig 3). Overall, our functional blocking experiments demonstrate that Src activity is not only necessary to stimulate lumen and tube formation, but is also necessary to maintain tubes that have already formed. In contrast, blockade of Src kinases fails to block EC sprouting behavior and the number of EC tip cells is actually significantly increased (Figs 3 and 4).

### The Src family kinases, Src, Fyn, and Yes, control EC lumen formation

**Fig 5:**
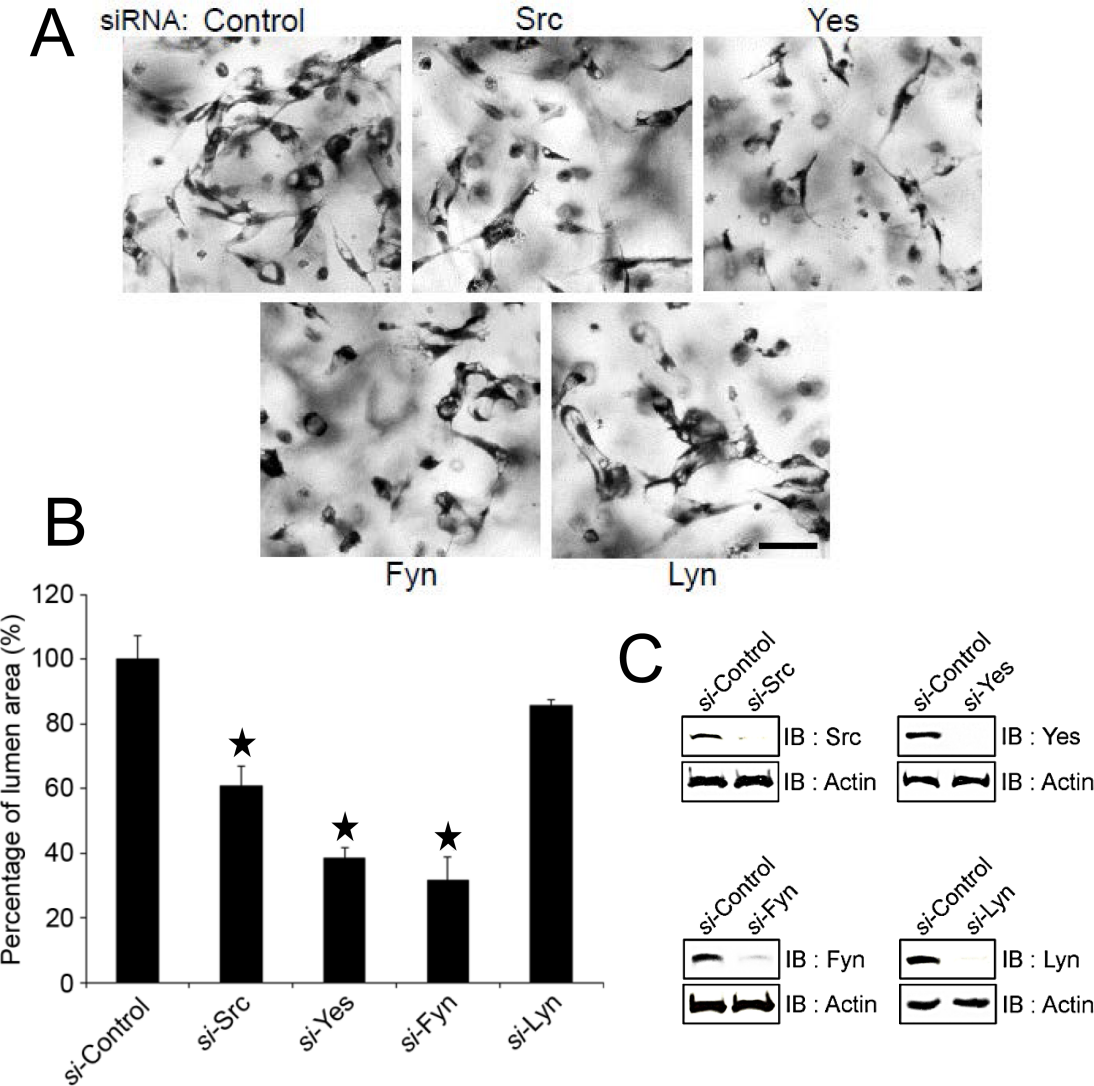
siRNA suppression of Src, Fyn and Yes, leads to blockade of endothelial lumen formation. ECs were treated with the indicated siRNAs and were assayed for their ability to form lumens. Cultures were fixed, stained, photographed (A) and quantitated for tube area (B). Data are reported as the mean total vessel area per high-power field (HPF) ± standard deviation (SD) (*n* = 15, p < 0.01). Asterisks indicate significance compared to control cultures. Western blots were performed to assess knockdown of the indicated Src family kinases with respect to a control siRNA. Bar equals 100 μm.

The PP2 inhibitor blocks multiple SFKs, and to address the role of individual SFKs that affect EC lumen and tube formation, we performed siRNA suppression experiments to knockdown each of the four major SFKs that are expressed by ECs which are Src, Fyn, Yes, and Lyn. Our results indicate that siRNA suppression of Src, Fyn, and Yes, all resulted in blockade of lumen formation while Lyn knockdown did not affect the process (Fig 5). This data indicates that there is some degree of redundancy among the SFKs with regard to the molecular control of vascular lumen and tube formation.

**Fig 6:**
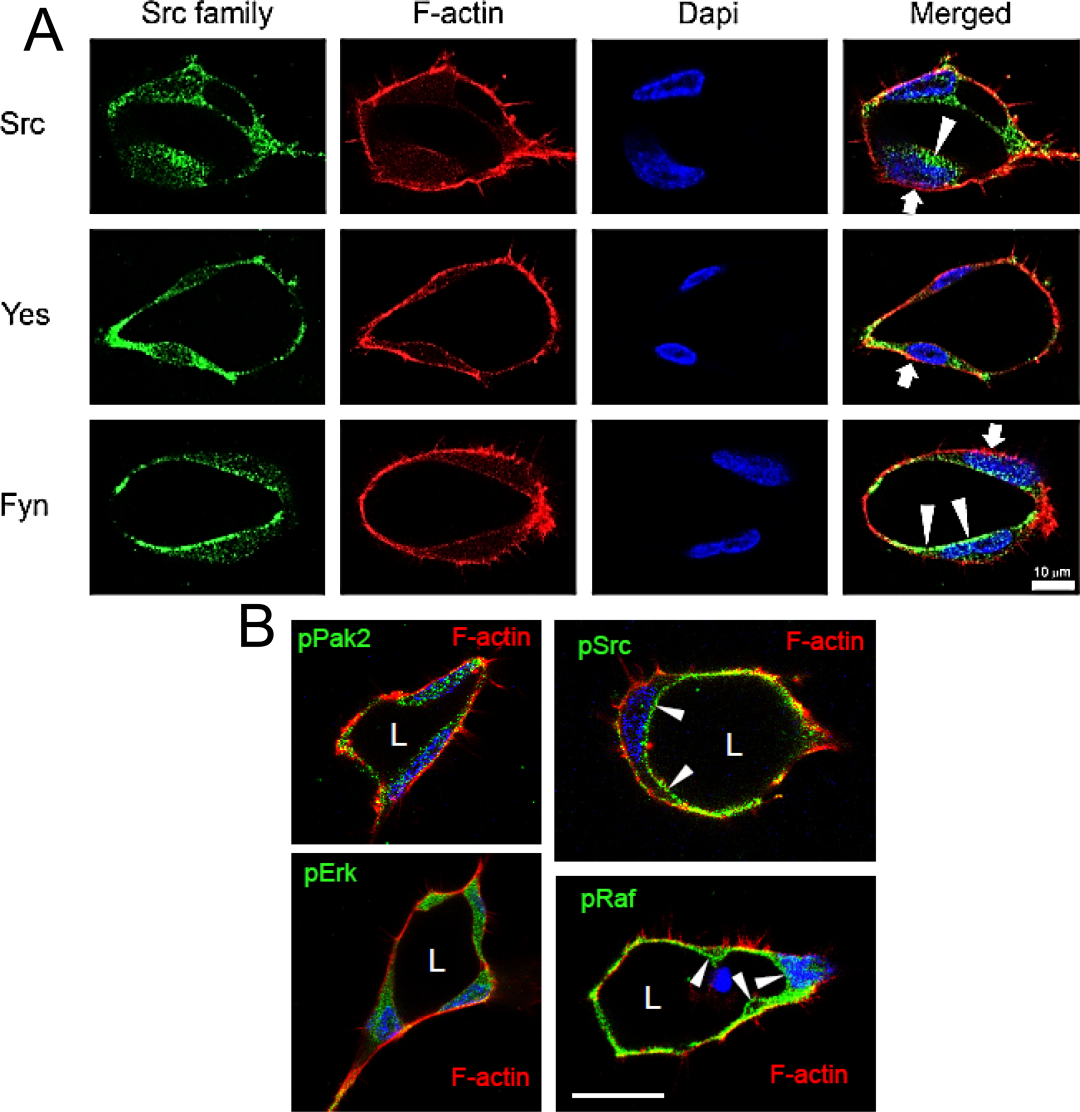
Fyn and Src target to the apical membrane surface during EC lumen formation. (A) ECs were allowed to form lumens and after 24 hr, were immunostained with antibodies specific for Src, Yes or Fyn. ECs were counterstained with phalloidin to label F-actin and Hoescht to label nuclei. Arrowheads indicate apical staining of Fyn or Src while arrowheads indicate the basal staining of F-actin. (B) ECs with lumens were immunostained with the indicated phospho-antibodies. Arrowheads indicate apical staining of phospho-Src (Y416) and phospho-c-Raf (Y314). L indicates an EC luminal space. Bar equals 25 μm.

We next addressed whether subcellular localization of these key SFKs are polarized to control the lumen formation process. We immunostained EC cultures with antibodies specific to these molecules which revealed that Fyn appears to be the most polarized showing an apical distribution (Fig 6A). Src also appears to be more localized in the apical/subapical domain, while Yes appears to be widely distributed. F-actin is localized to the basal domain. As shown with phosphotyrosine, phospho-Src staining reveals marked localization to the apical surface (Fig 6B). Interestingly, Src is known to activate c-Raf to participate in controlling EC tube morphogenesis [13]. Using an antibody specific to a known Src phosphorylation site on Raf, pY341Raf, we showed that this modified form of c-Raf is also localized at the developing apical surface (Fig 6B). By contrast, other key kinases known to control lumen formation including pPak2 and pErk [12, 13] are observed in an even distribution within ECs.

### Activated phospho-Src is markedly polarized in apical membranes during EC lumen and tube formation

**Fig 7:**
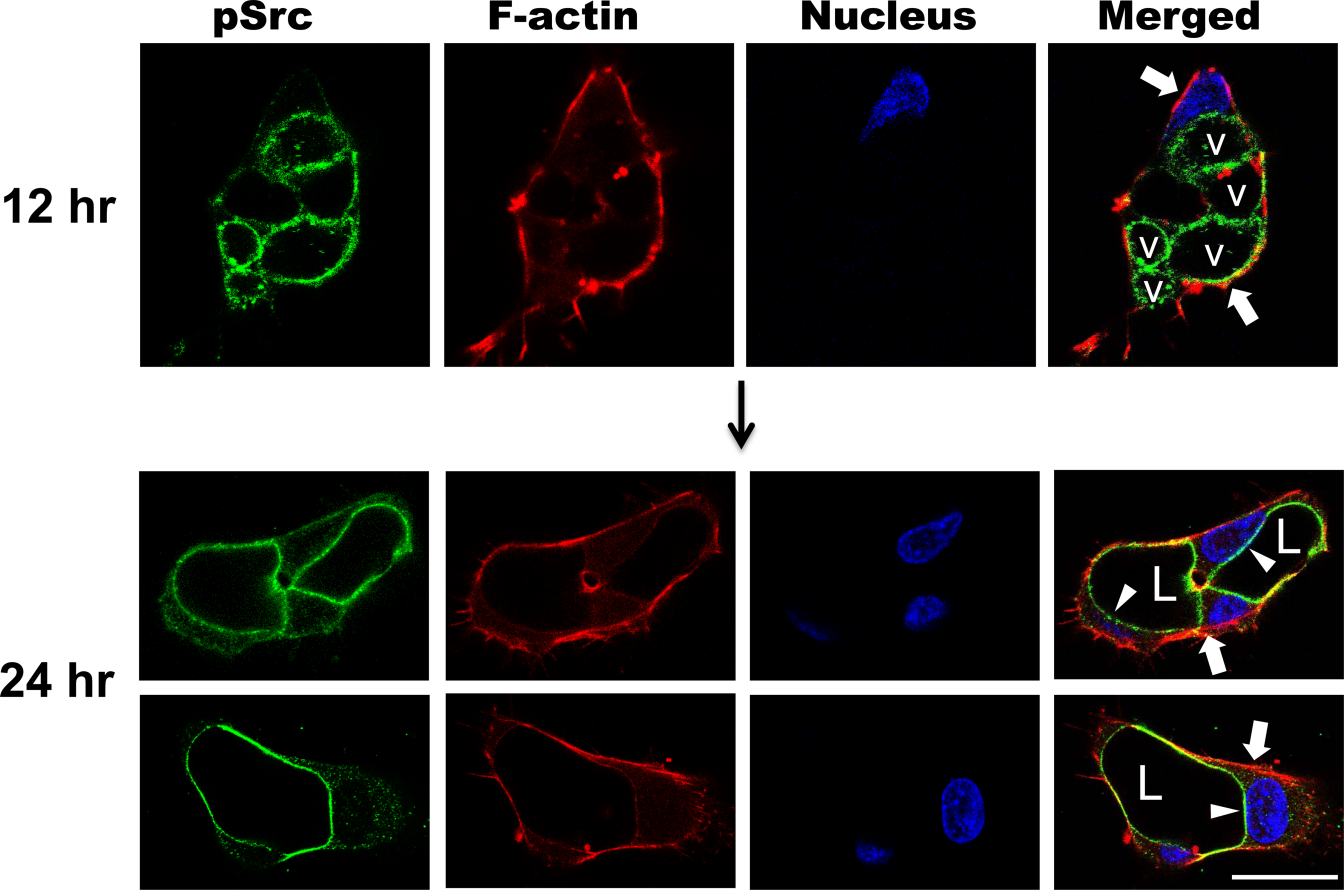
Marked apical membrane targeting of activated Src kinases during EC lumen formation and multicellular EC tube assembly. ECs were allowed to form lumen structures for either 12 or 24 hr and after fixation were stained for phospho-Src (Y416). Arrowheads indicate marked staining of the apical surface, while arrows indicate the basal staining of pattern of F-actin (labeled with phalloidin). L indicates lumens while v indicates vacuoles. Bar equals 25 μm.

**Fig 8:**
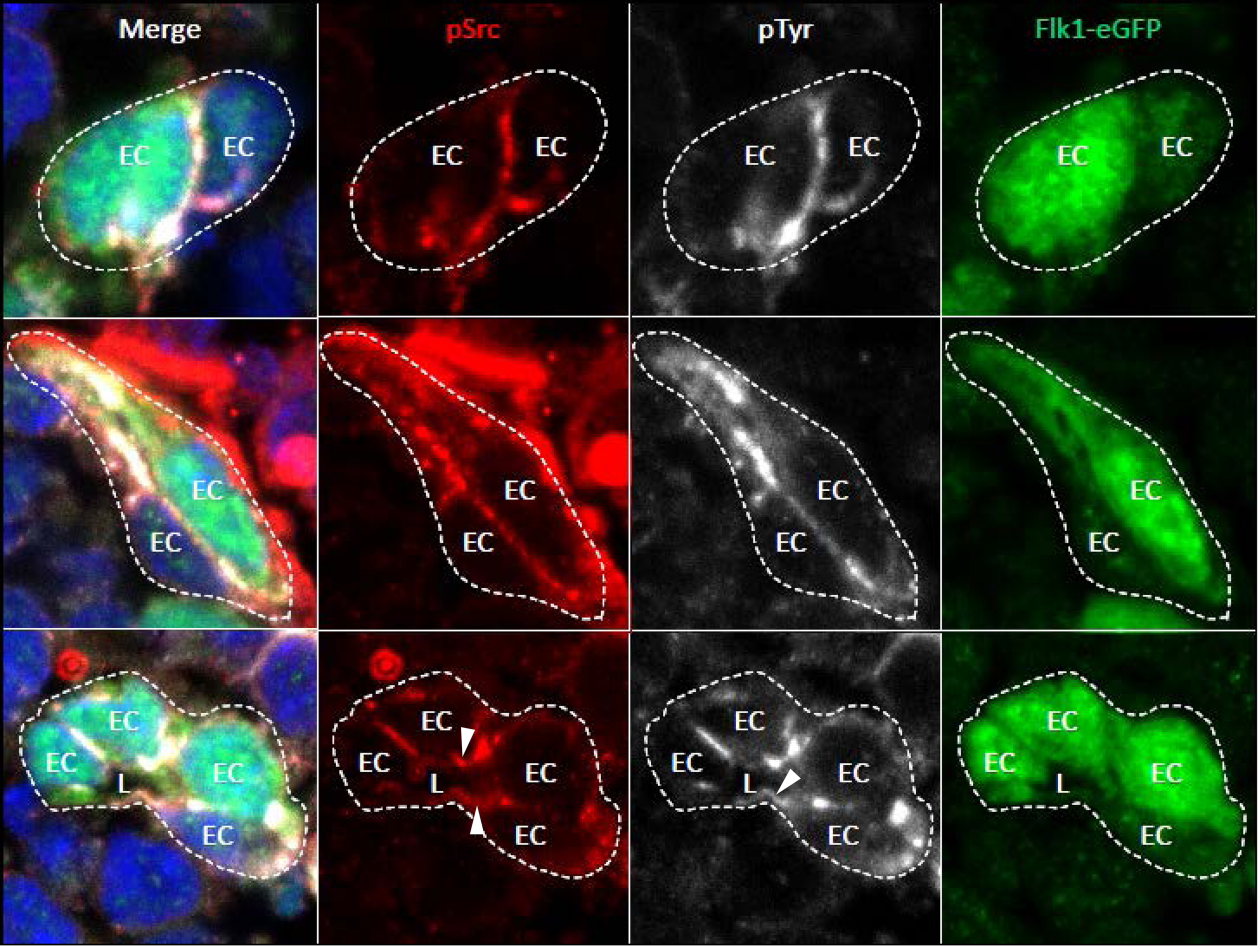
Labeling of the EC apical membrane surface during lumen formation in vivo with anti-phosphotyrosine and phospho-Src antibodies. Mouse yolk sac embryonic vessels expressing GFP within ECs were fixed at E8 and immunostained with anti-phosphotyrosine and anti-phospho-Src antibodies. Arrowheads indicate staining of phosphotyrosine or phospho-Src at the apical membrane surface as these embryonic mouse vessels establish their luminal space (lower panels).

To address the distribution of activated Src kinases during EC lumen formation, we immunostained EC cultures at different times of morphogenesis to examine the distribution of phospho-Src in relation to other polarized structures including F-actin and acetylated tubulin (Figs 7 and 8). Strong localization of phospho-Src is observed within intracellular vacuoles over time, and then in the apical membrane after vacuoles fuse together to form the lumen compartment (Fig 7). The apical membrane is delineated in a remarkable fashion using phospho-Src antibodies at the 24 hr time point (Fig 7). In addition, we observe similar apical membrane staining in more mature EC-lined tubes with or without the presence of pericytes after 120 hr of culture (S1 Fig). We next asked whether we observe similar phosphotyrosine and phospho-Src staining in the nascent apical membrane during EC lumen formation *in vivo*. We stained the developing mouse yolk sac vasculature at E8 at the time when we begin to observe lumen formation in this vasculature (Fig 8). Our immunostaining does reveal increased phosphotyrosine and phosphotyrosine staining at the apical surface during mouse EC lumen formation *in vivo* (Fig 8).

### Activated Src co-distributes with Rac1, a key regulator of EC lumen and tube formation

**Fig 9:**
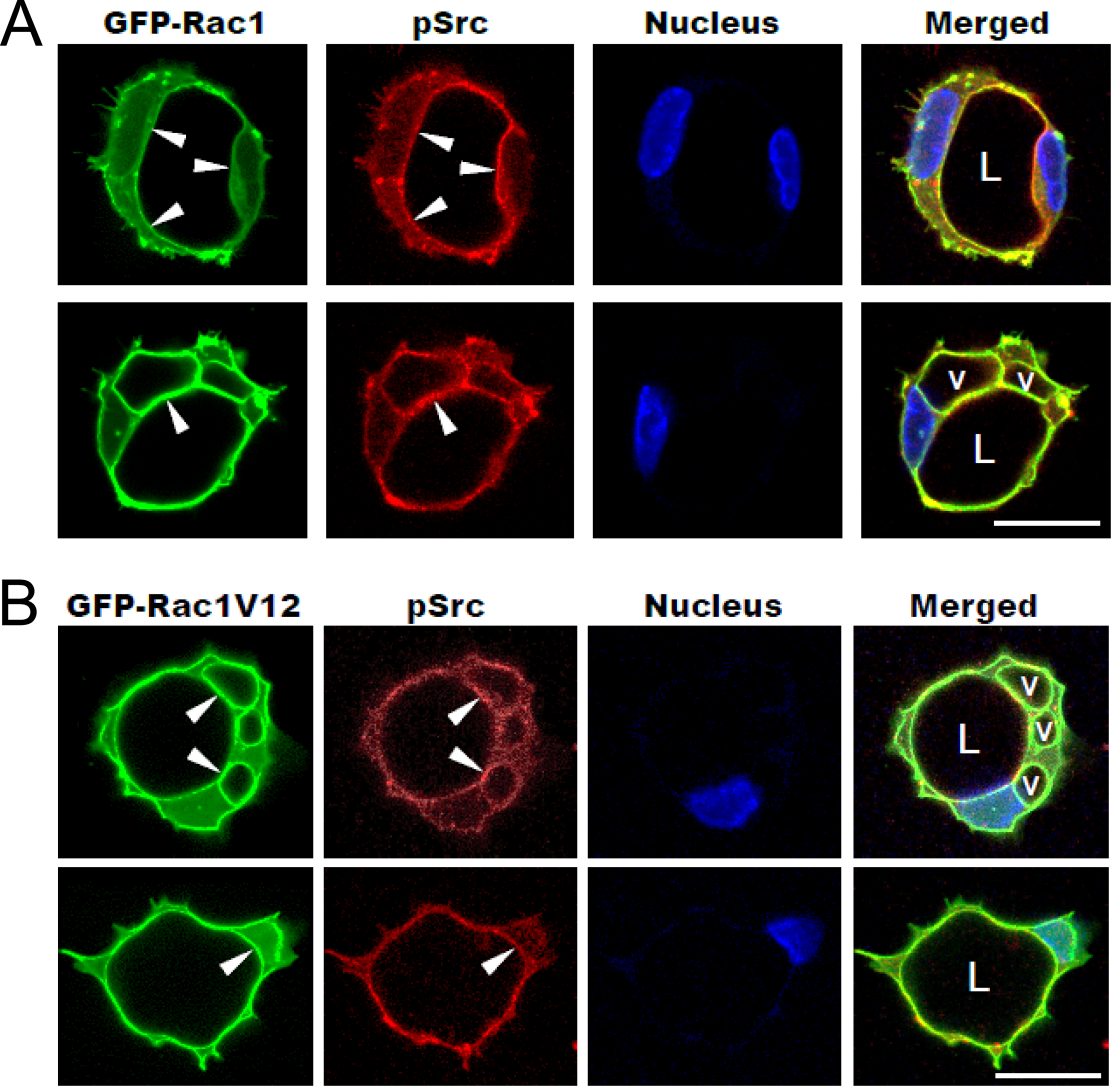
Co-distribution of GFP-Rac1 with phospho-Src at the apical membrane surface during EC lumen formation. ECs were induced to express GFP-Rac1 and were allowed to form lumens for 12 hr (A) or 16 hr (B) and then fixed. They were then immunostained to visualize phospho-Src. Arrows indicate overlapping GFP-Rac1 and pSrc staining at the apical membrane surface. L indicates lumen and v indicates vacuoles. Bar equals 25 μm.

Previous work from our laboratories reveals a critical role for the small GTPases, Cdc42 and Rac1 in EC lumen and tube assembly [11]. GFP-Rac1 was demonstrated to be recruited to intracellular vacuoles and the apical surface during EC lumen formation. Here, we demonstrate co-distribution of activated Src with GFP-Rac1 within intracellular vacuoles and the developing apical surface during EC lumen formation (Fig 9). Thus, activated Src along with the small GTPase Rac1, demarcates the vacuole and apical luminal membrane surface during these processes.

### Apical polarization of both phospho-Src and acetylated tubulin during EC lumen formation

**Fig 10:**
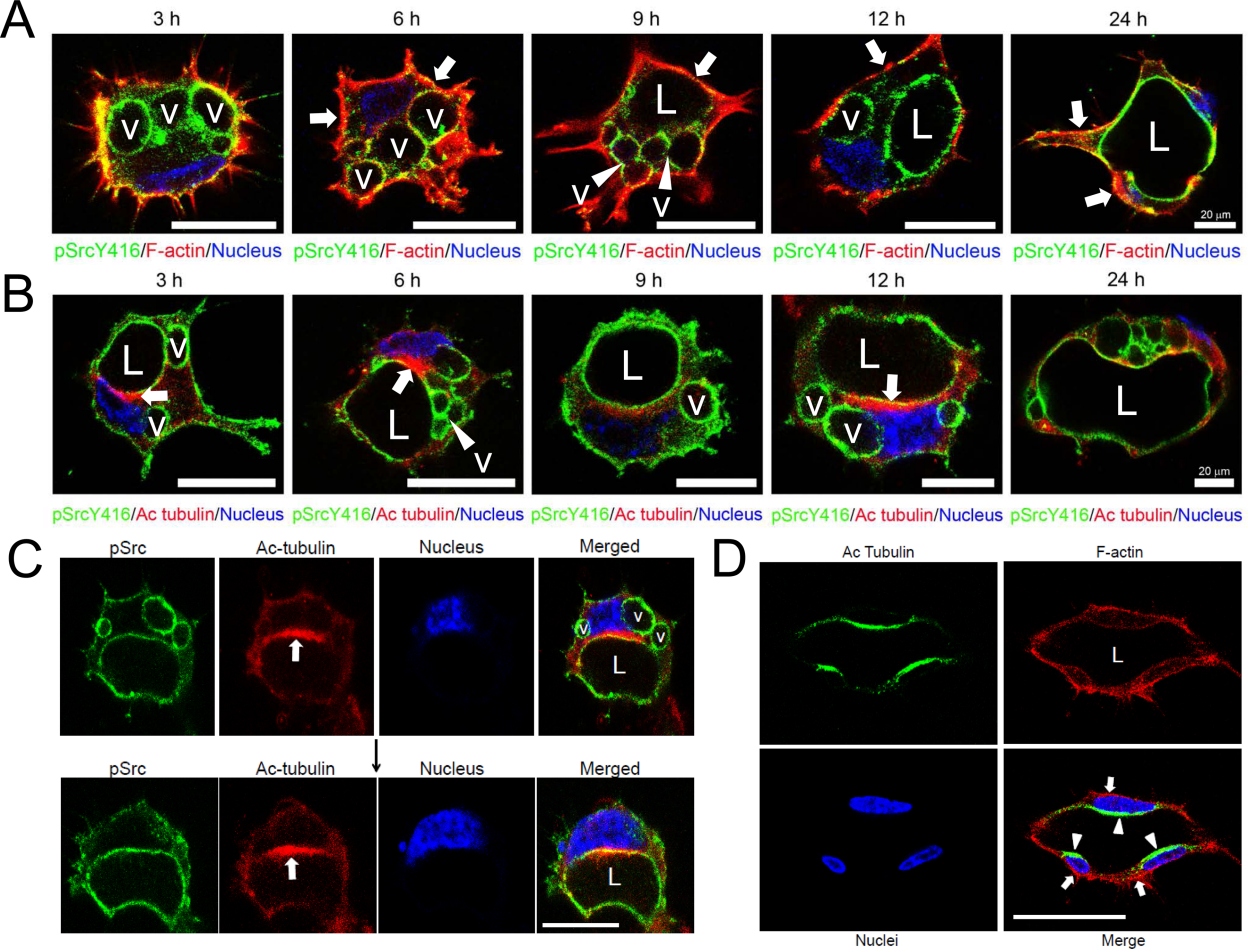
Apical and subapical targeting of phospho-Src and acetylated tubulin, respectively, to polarize and regulate EC lumen formation. (A, B) ECs were suspended to induce lumen formation and at the indicated times of culture, were fixed, and stained for either F-actin (A) or acetylated tubulin (B) Bar equals 20 μm. Arrows indicate vacuoles which stain strongly with phospho-Src antibodies, while arrowheads indicate basal F-actin staining (A) or subapical acetylated tubulin staining (B). (C) Vacuoles are targeted along tubulin tracks labeled with acetylated tubulin to delineate the apical membrane surfaces which are labeled with phospho-Src antibodies. Upper panels are images derived from 12 hr cultures while the lower panels derive from 16 hr cultures. Bar equals 25 μm. (D) Multicellular lumen structure demonstrating subapical targeting of acetylated tubulin and basal targeting of F-actin. Bar equals 50 μm.

More detailed *in vitro* time courses were performed to assess the relationship between the developing apical membrane and the polarized underlying cytoskeleton during EC lumen formation. We examined the distribution of pSrc, acetylated tubulin (which is highly localized in a subapical domain and surrounds intracellular vacuoles), and F-actin which is predominantly observed basally (Fig 10A, 10B and 10D). We hypothesize that the tubulin cytoskeleton (which is strongly acetylated during lumen formation) directs vacuole transport and fusion in a polarized, perinuclear manner. Acetylated tubulin is observed to be highly localized in areas where pSrc-labelled vacuoles appear to be fusing together over time to form a polarized lumen compartment (Fig 10B and 10C). This data demonstrates an important role for coordinated apical membrane and cytoskeletal polarization during EC lumen and tube formation.

### Src-GFP and Fyn-GFP fusion proteins label intracellular vacuole and apical membranes during EC lumen formation

**Fig 11:**
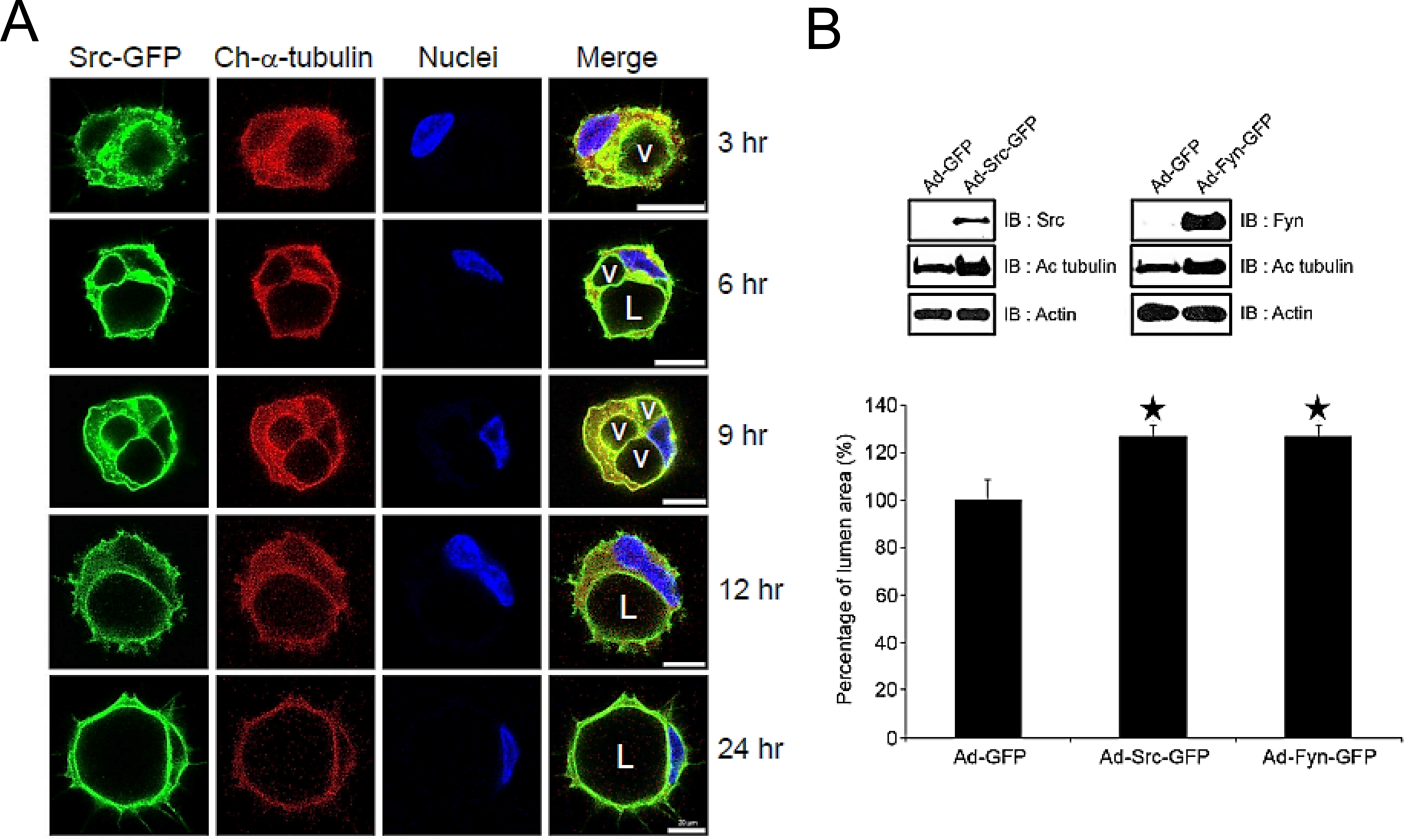
Apical targeting of Src-GFP and stimulation of EC lumen formation by increasing expression of Src or Fyn. ECs were induced to express either Src-GFP (A, B) or Fyn-GFP (B) and were induced to form lumens. In some cultures, mCherry-α-tubulin was co-expressed along with Src-GFP (A). Cultures were fixed at the indicated time points and were imaged using confocal microscopy. L indicates lumens while v indicates vacuoles. Bar equals 20 μm. (B) Src-GFP, Fyn-GFP or GFP alone were expressed in ECs and were then seeded to form lumens. Data are reported as the mean total vessel area per high-power field (HPF) ± standard deviation (SD) (*n* = 15, p < 0.01). Asterisks indicate significance compared to control cultures.

**Fig 12:**
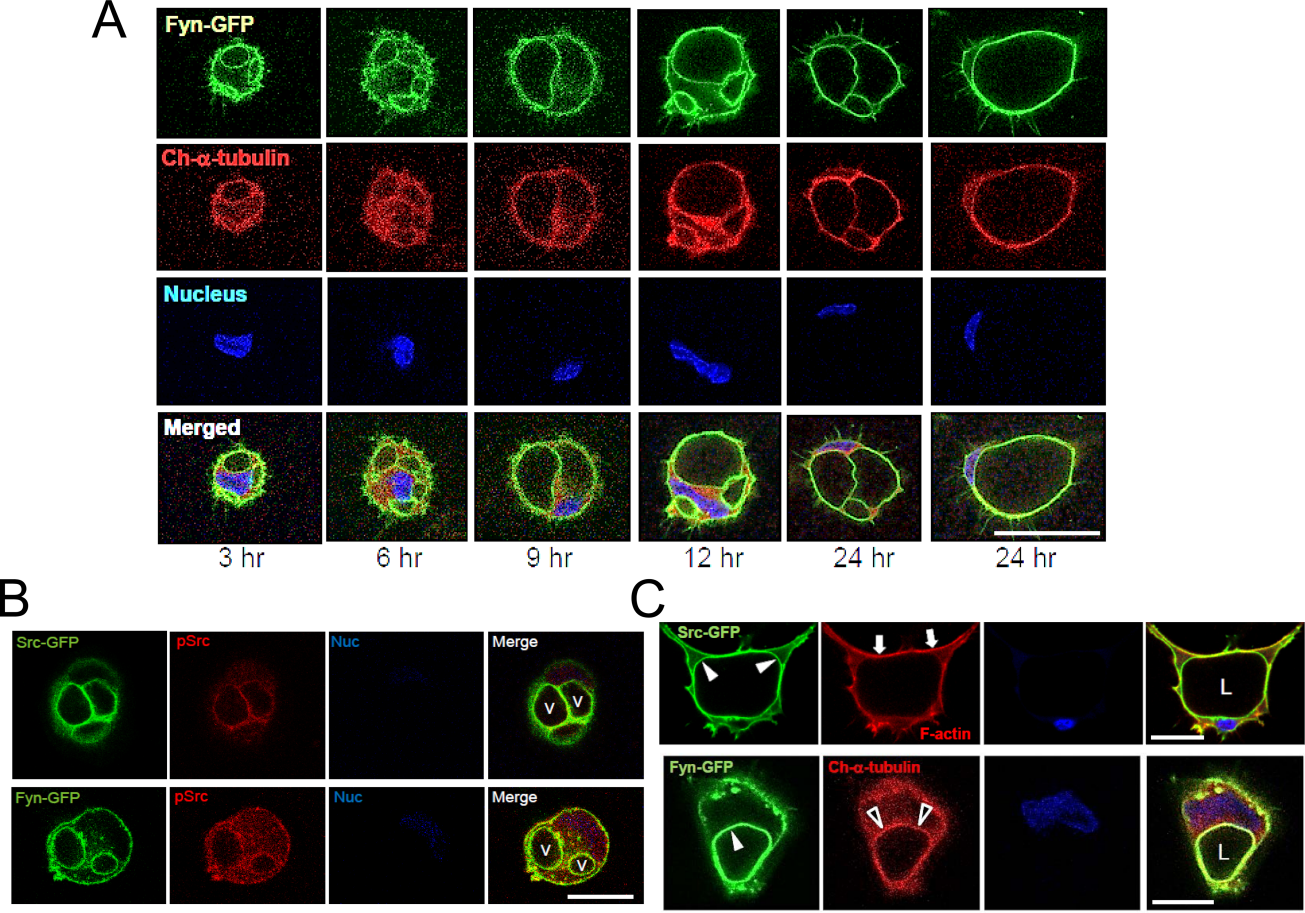
Marked apical targeting of Fyn-GFP and Src-GFP during EC lumen formation. (A) Fyn-GFP was expressed in ECs (along with mCherry-α-tubulin) and lumen formation was examined over time using confocal microscopy. Bar equals 50 μm. (B) Targeting of Fyn-GFP and Src-GFP with co-localized phospho-Src in intracellular vacuoles during EC lumen formation. Vacuoles are indicated by v. Bar equals 25 μm. (C) Fyn-GFP and Src-GFP target to the apical membrane surface, while Cherry-α-tubulin strongly underlies Fyn-GFP in a subapical distribution while F-actin is localized in a basal position. L indicates lumen. White arrowheads indicate the apical membrane surface; black arrowheads indicate subapical/apical positioning of α-tubulin. Arrows indicate the predominant basal distribution of F-actin. Bar equals 50 μm in the upper panel and 25 μm in the lower panel.

**Fig 13:**
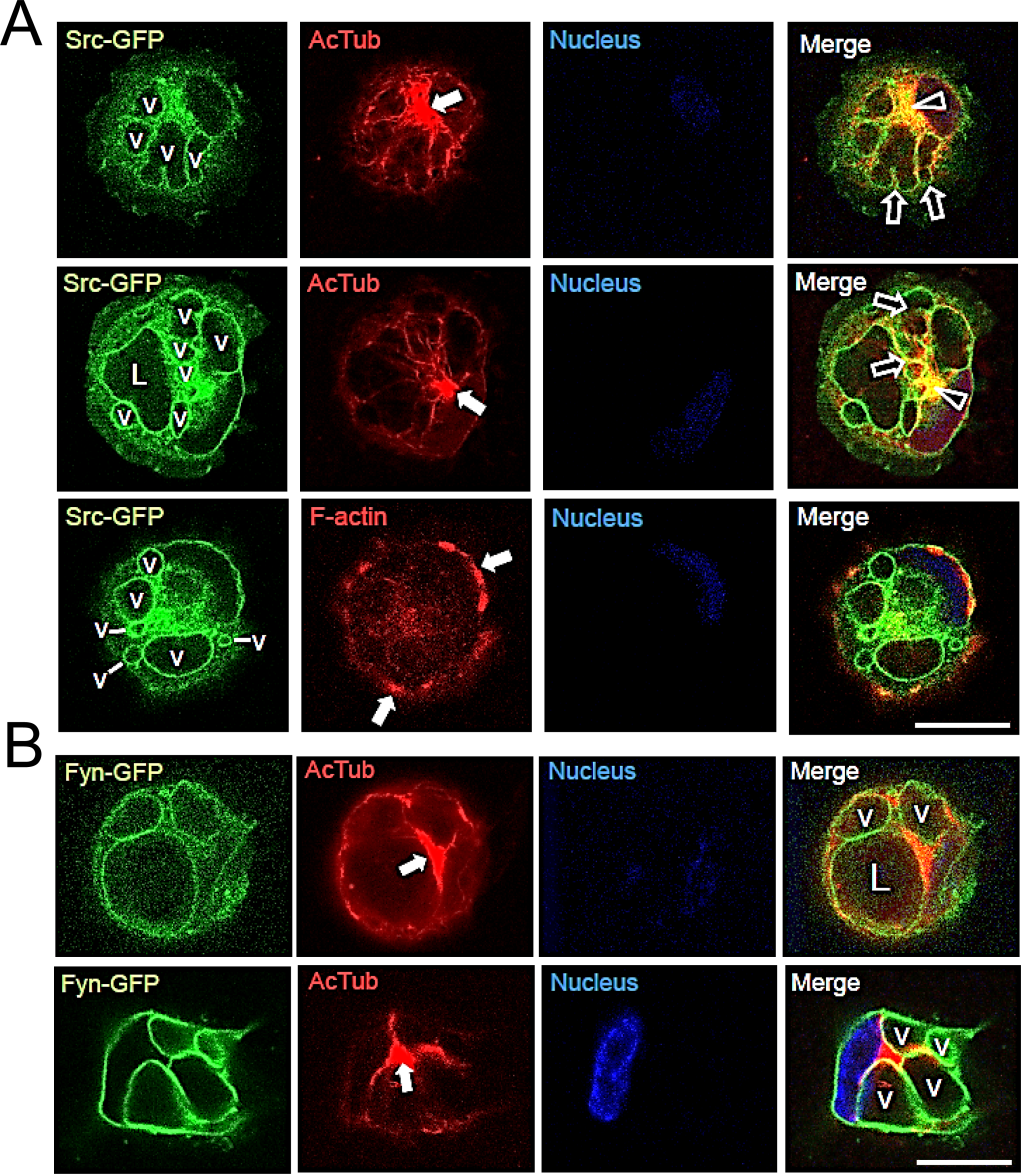
Src-GFP and Fyn-GFP, which are localized in vacuole membranes, are contacted by acetylated tubulin tracks that direct polarized fusion events to generate the apical membrane during EC lumen formation. Src-GFP and Fyn-GFP were expressed in ECs and these cells were suspended in gels to allow for vacuole and lumen formation. (A) After 4 hr of culture, collagen gels were digested with collagenase and cells undergoing lumen formation were rapidly attached to glass coverslips. After fixation, ECs were stained for acetylated tubulin (arrowheads) or F-actin (white arrows). Black arrows indicate vacuole-vacuole fusion leading to the development of the apical membrane and luminal surface. L indicates lumen and v indicates vacuoles. Bar equals 25 μm. (B) ECs expressing Fyn-GFP were allowed to form lumens for 16 hr, were fixed and stained for acetylated tubulin (arrows). Bar equals 25 μm.

An alternative approach to examine the distribution of SFKs during EC lumen and formation was to tag them with GFP and to express them in ECs during morphogenic events. To localize tubulin within the same cells, we co-expressed mCherry-α-tubulin and evaluated EC lumen formation over time (Figs 11 and 12). Both Src-GFP and Fyn-GFP accumulate in intracellular vacuole and apical lumen membranes during EC lumen formation consistent with our immunostaining results with SFKs, phospho-Src, and phosphotyrosine antibodies. The Src and Fyn containing vacuoles accumulate in a polarized, perinuclear region which is surrounded by an acetylated tubulin cytoskeletal apparatus (Fig 13). Interestingly, mCherry-α-tubulin also accumulates underneath the apical membrane surface to support the lumen formation process, which is particularly pronounced when co-expressed with Fyn-GFP (Fig 12A and 12C). Of the SFKs, Fyn is able to bind to tubulin [47], a critical regulator of EC lumen formation and apical-basal polarity [21]. To query a functional role for these kinases, we increased the expression of either Src or Fyn in ECs and show that both kinases markedly enhance lumen formation (Fig 11B). Overall, using multiple approaches, we demonstrate that SFKs, particularly, Src, Fyn, and Yes, play a fundamental role in controlling EC lumen and tube formation through the development, maturation, and maintenance of the EC apical surface.

## Discussion

In this work, we have investigated and identified a key role for Src family kinases in regulating the development of the apical membrane domain during EC lumen and tube formation. This work adds to recent studies from our laboratory showing fundamental roles for the small GTPases, Cdc42, k-Ras, Rap1b and Rac2 in apical membrane development and polarization during EC lumen formation [11, 24, 38]. Here, we demonstrate that activated Src kinases are recruited to the apical membrane surface during EC lumen formation and functional blockade of these kinases markedly inhibits this process. This can be demonstrated by immunostaining with anti-phosphotyrosine and anti-phospho-Src antibodies (which recognize multiple activated Src isoforms). Marked and highly selective expression is seen on intracellular vacuole membranes as well as the apical surface during EC lumen formation *in vitro* and *in vivo*. Furthermore, we have demonstrated that Src kinases control EC lumen formation during the two major processes that regulate vascular tube morphogenesis which are vasculogenesis and angiogenesis. In assays that mimic both processes, we demonstrate that Src kinase activity is necessary to form as well as maintain the luminal compartment in networks of EC-lined tubes, whether this results from vasculogenic multicellular EC assembly or following EC sprouting events. Finally, we demonstrate that multiple Src kinases play a role in these events including Src, Fyn, and Yes, and that these participate along with other key regulators of EC polarity including small GTPases as well as both the microtubule and actin cytoskeletons.

Major progress has occurred over the past 20 years in our understanding for how ECs form lumens and tubes during vascular morphogenesis [1, 4–7]. Defined *in vitro* systems using human ECs have contributed substantially to our understanding of these processes [1, 44, 48, 49]. In numerous instances, the information has been recapitulated *in vivo* [14, 17, 25, 26, 28, 50, 51] and in a number of cases, vascular morphogenic defects detected *in vivo* have been recapitulated *in vitro* [17, 18, 23, 24, 52]. This rigorous dual approach is being increasingly employed as the major approach to identify and characterize the fundamental molecules and signaling pathways that are necessary for ECs to form lumen and tube networks.

During vascular morphogenesis, ECs become polarized and are exposed to fluid on their apical surface and extracellular matrix on their basal surface. It has been challenging to identify highly polarized molecules that selectively label the apical surface during EC lumen formation in contrast to epithelial lumen formation where this polarization is more readily observed. In recent studies, we have described marked cytoskeletal polarization during EC lumen formation, where we have demonstrated the subapical distribution of acetylated tubulin which supports the apical membrane and strong basal distribution of F-actin (correlating with areas of EC-extracellular matrix contacts) [21, 24, 38]. We have also demonstrated the ability of small GTPases and their effectors to target the apical membrane surface including Rac1, k-Ras, and Rap1b, as well as the Ras and Rap effector, Rasip1 [24]. In addition, we have shown the subapical accumulation of Cdc42, which can be observed to co-localize with acetylated tubulin in polarized, perinuclear domains that control vacuole localization and fusion events to direct the lumen formation process [24, 38]. Our work shows that these small GTPases direct both membrane trafficking and polarization in conjunction with cytoskeletal polarization events (in particular, subapical acetylated tubulin) to regulate vesicular trafficking and fusion to direct EC lumen and tube assembly.

**Fig 14:**
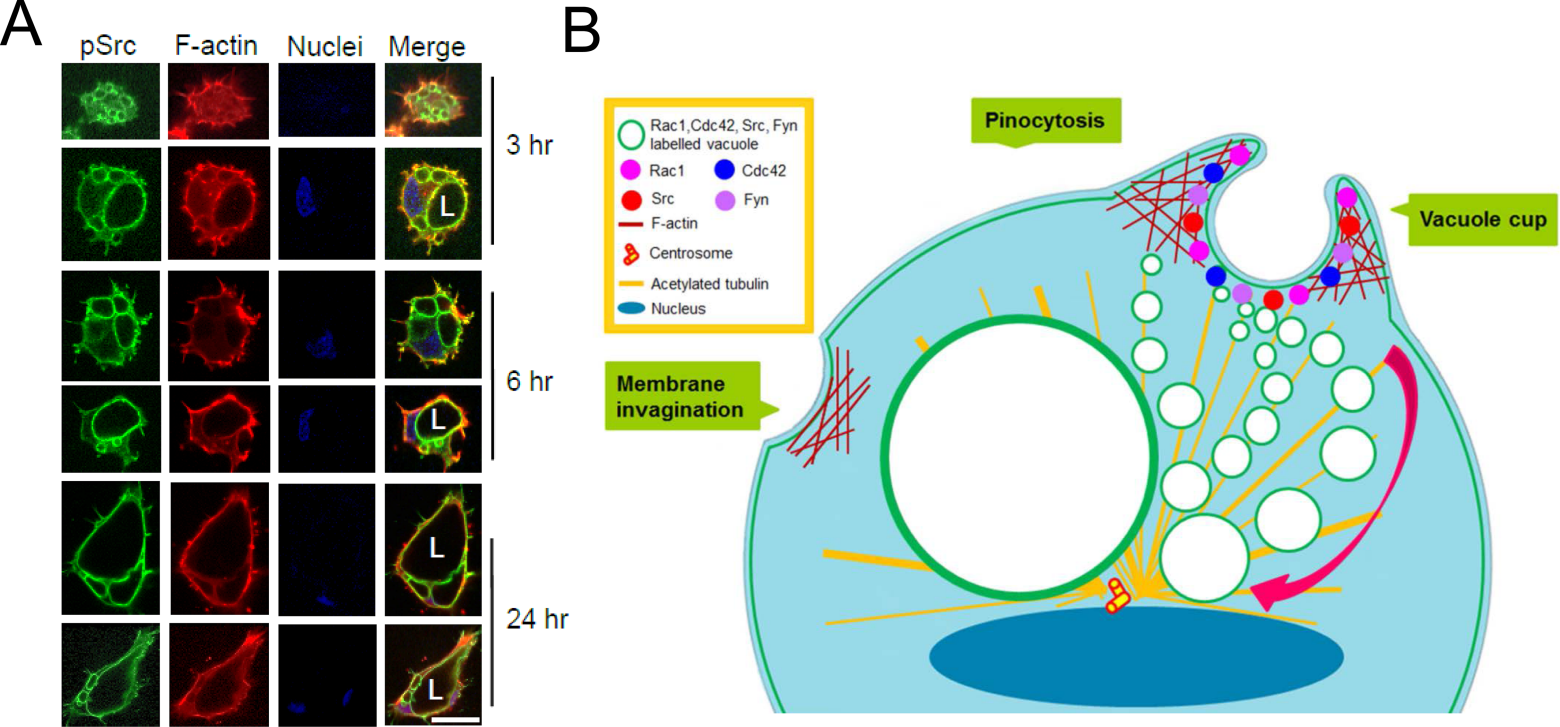
Active Src containing vacuoles traffic and fuse along the subapically polarized microtubule cytoskeleton enriched in acetylated tubulin to develop the apical membrane surface during endothelial lumen formation. (A) Time course of EC lumen formation reveals marked accumulation of activated Src in vacuoles (v) which fuse together in a subapical, polarized and perinuclear region to create the luminal (L) compartment. Bar equals 25 μm. (B) Schematic diagram illustrating how pinocytic intracellular vacuoles enriched in phospho-Src kinases accumulate and are transported along polarized microtubule cytoskeletal tracks to fuse together in a perinuclear region to form the EC luminal space.

Our findings here add to this previous work by reporting the targeting of activated Src kinases to the apical surface of ECs forming lumens. We show this via the marked presence of phosphotyrosine-containing proteins and phospho-Src kinases in intracellular vacuoles and the apical surface as these vacuoles fuse together to form the EC luminal compartment over time (Fig 14). The staining that we observe delineates the developing apical compartment in a dramatic fashion revealing that considerable membrane polarity exists even during the earliest stages of EC lumen formation and that it persists after the apical and luminal surface is established. Importantly, we demonstrate the functional relevance of this apical targeting of activated Src kinases. Pharmacologic blockade or siRNA suppression of Src kinases strongly inhibits the EC lumen formation process during either vasculogenic or angiogenic tube assembly. Blockade of Src kinases after EC lumen and tube formation has occurred results in tube collapse suggesting that they are necessary for lumen maintenance.

Another critical aspect of the lumen formation cascade is the polarization of the cytoskeleton and we demonstrate here how activated Src containing vacuoles/ vesicles can be shown to be surrounded and likely guided by the microtubule cytoskeleton which is highly polarized and enriched with acetylated tubulin. Both the microtubule and actin cytoskeletons are required for EC lumen and tube formation and are regulated by key signaling molecules including Src kinases and small GTPases such as Cdc42, k-Ras, Rac, and Rap1 [11, 12, 24, 25]. Previous work from our laboratory revealed a major role for the microtubule associated proteins, EB1, p150glued and Clasp-1, which are all necessary for microtubule assembly and tubulin acetylation during lumen formation, possibly through suppression of tubulin deacetylase activity [21]. These microtubule regulatory proteins contribute toward the cytoskeletal polarization necessary to direct membrane trafficking (decorated with Src kinases, and small GTPases such as Rac and k-Ras) to a polarized position to direct the creation of a specific apical membrane domain during the EC lumen formation process. The kinase signaling cascades that we have identified including Src, Pak, Raf, and Erk are necessary for polarization events to occur at both the level of the cytoskeleton as well as apical membrane trafficking [13]. The EC lumen and tube formation process also requires integrin and membrane-type matrix metalloproteinase (specifically MT1-MMP) activity which are necessary together to control these fundamental polarization events [16, 39]. Future studies need to address whether key vesicular trafficking molecules such as Rab GTPases control these processes as well and how these molecules interact with other key regulators to affect EC lumen formation. A major challenge is to elucidate how and when these regulators of lumen formation act to control the major stages of this process. Overall, our findings here illustrate how a key kinase family (i.e. Src, Fyn, and Yes) controls apical membrane polarization during EC lumen and tube formation to fundamentally regulate vascular morphogenesis.

## Supporting information

S1 Checklist The arrive guidelines checklist. (DOCX)

S1 Fig Inhibition of Src leads to increased EC tip cells during sprouting events. (A) ECs were allowed to sprout for 24 hrs in the presence or absence of the Src inhibitor, PP2, using a defined growth factor model system in 3D collagen matrices. After 24 hr, cultures were fixed and stained with antibodies to the EC tip cell markers, CXCR4 or ESM-1. The gels were cross-sectioned and the monolayer surface is indicated by the arrows. Arrowheads indicate invading EC tip cells. Bar equals 100 μm. (B) Activated Src and phospho-tyrosine staining are observed in an apical membrane position in more mature capillary tubes in 3D collagen matrices. EC only or EC-pericyte (mCherry-labeled) co-cultures were fixed after 120 hr and were stained with either phospho-Src (pSrc) or phospho-tyrosine (pTyr) antibodies. Arrowheads indicate apical membrane staining, while L indicates lumen. Arrows indicate pericytes. Bar equals 50 μm.
(TIF)
