# Supplementary figures and images for: Src- and Fyn-dependent apical membrane trafficking events control endothelial lumen formation during vascular tube morphogenesis
Source: PLoS One. 2017 Sep 14;12(9):e0184461. doi: 10.1371/journal.pone.0184461 (PMC5598984; doi:10.1371/journal.pone.0184461)

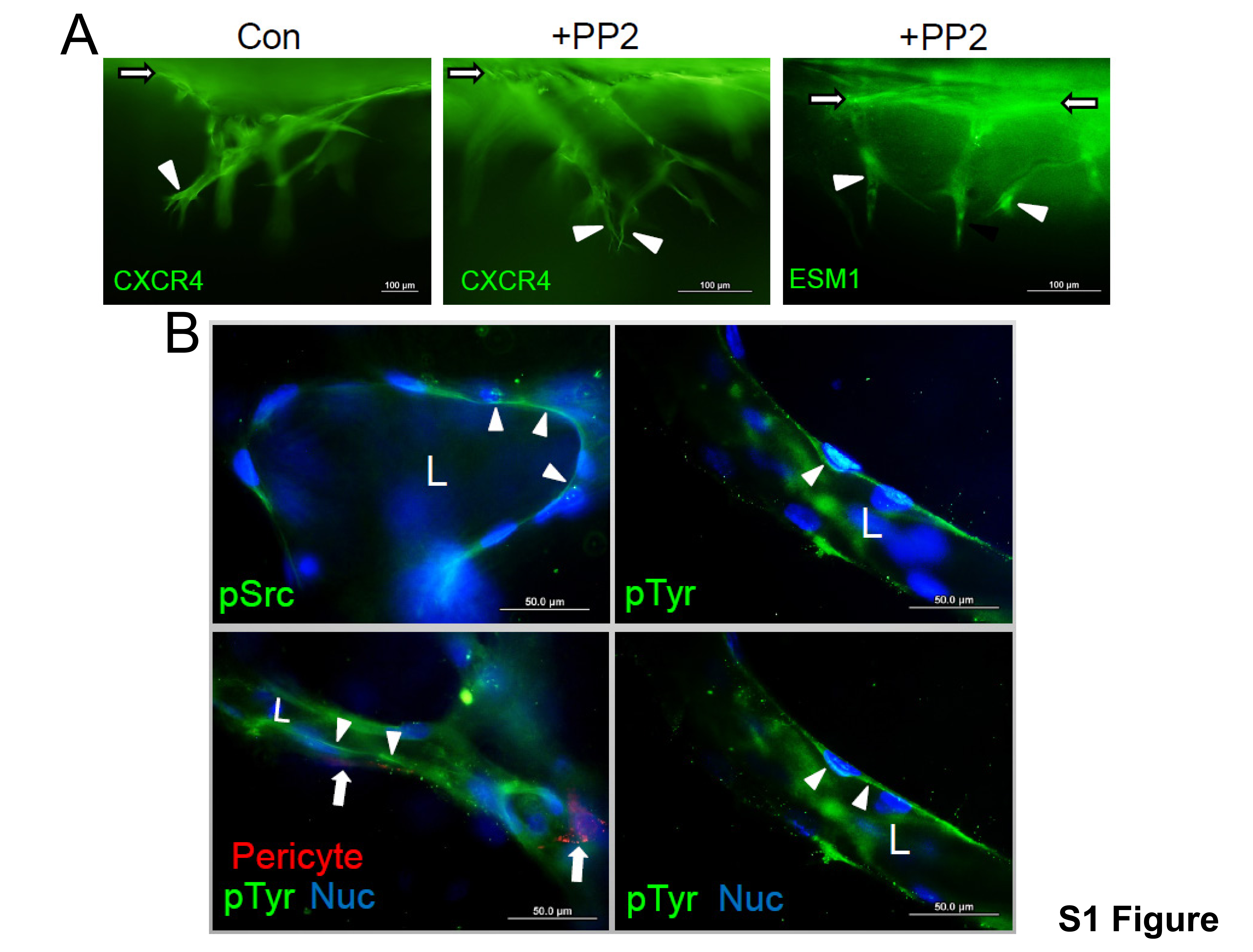

Supplement: S1 Fig — (A) ECs were allowed to sprout for 24 hrs in the presence or absence of the Src inhibitor, PP2, using a defined growth factor model system in 3D collagen matrices. After 24 hr, cultures were fixed and stained with antibodies to the EC tip cell markers, CXCR4 or ESM-1. The gels were cross-sectioned and the monolayer surface is indicated by the arrows. Arrowheads indicate invading EC tip cells. Bar equals 100 μm. (B) Activated Src and phospho-tyrosine staining are observed in an apical membrane position in more mature capillary tubes in 3D collagen matrices. EC only or EC-pericyte (mCherry-labeled) co-cultures were fixed after 120 hr and were stained with either phospho-Src (pSrc) or phospho-tyrosine (pTyr) antibodies. Arrowheads indicate apical membrane staining, while L indicates lumen. Arrows indicate pericytes. Bar equals 50 μm. (TIF) [file pone.0184461.s002.tif]
